# Crizotinib: A Breakthrough for Targeted Therapies in Lung Cancer

**DOI:** 10.6004/jadpro.2012.3.4.8

**Published:** 2012-07-01

**Authors:** Jennifer Kwon, ALISON MEAGHER

**Affiliations:** From Roswell Park Cancer Institute, Buffalo, New York

Lung cancer is the leading cause of cancer-related mortality in the United States, with approximately 226,160 new cases of lung and bronchial cancer predicted to be diagnosed in 2012, along with approximately 160,340 deaths due to the disease (Siegel, Naishadham, & Jemal, 2012). Worldwide, approximately 1.6 million new cases of lung cancer are diagnosed every year, and 1.38 million deaths were recorded in 2008 (Ferlay et al., 2010).

The majority of non–small cell lung cancer (NSCLC) patients are diagnosed with advanced disease, and many patients diagnosed with early-stage disease will have recurrence or develop metastatic disease. Only about 15% of all patients with lung cancer are alive at 5 years or more after diagnosis (National Comprehensive Cancer Network [NCCN], 2011). Early diagnosis and expanded treatment options for lung cancer continue to be a challenge for improving outcomes.

Current treatment approaches include surgical resection, chemotherapy, and radiation therapy. Chemotherapy regimens are usually reserved for advanced stages of lung cancer in adjuvant or neoadjuvant settings. Non–small cell lung cancer tumors, however, are not sensitive to most chemotherapy regimens, with average response rates ranging from 25% to 30% (NCCN, 2011). New treatment options, both in the areas of nonspecific cytotoxic chemotherapy and targeted therapies, are needed to improve survival in patients with advanced stages of NSCLC.

Advances in understanding tumor biology and oncogenes have helped identify several molecular targets in the treatment of NSCLC. Targeted therapies affect proteins that are involved with tumor cell proliferation and lead to growth inhibition and apoptosis. For example, erlotinib (Tarceva), an epidermal growth factor receptor (EGFR) inhibitor, produced responses in a group of patients with NSCLC who were positive for EGFR mutations (Kwak et al., 2010). These findings suggest that patient outcomes can be improved by testing tumor markers for specific mutated pathways and targeting treatment against those pathways.

## The *EML4-ALK* Gene

Mutations or translocations of the anaplastic lymphoma kinase (*ALK*) gene have been detected in several types of cancer, including anaplastic large-cell lymphoma, neuroblastoma, myofibroblastic tumor, and NSCLC (Kwak et al., 2010). The *ALK* gene fused with the echinoderm microtubule-associated proteinlike 4 (*EML4*) gene was initially discovered in a Japanese patient with NSCLC (Sasaki et al., 2010b). The presence of *EML4-ALK* fusion is seen in an estimated 2% to 7% of all NSCLC cases (Kwak et al., 2010). This translates to about 10,000 patients in the United States being carriers of the gene. Patients with *EML4-ALK* rearrangements tend to be males who are younger, nonsmokers or light smokers, and who have adenocarcinomas (Kwak et al., 2010). In addition, patients with *EML4-ALK* rearrangements are generally mutually exclusive with EGFR and KRAS mutations (Takahashi et al., 2010).

The Vysis ALK Break Apart FISH Probe Kit, designed to identify ALK-positive NSCLC patients, is approved by the US Food and Drug Administration (FDA) to detect rearrangements of the *ALK* gene (Pfizer, 2011). The FISH probe appears to be better than immunohistochemistry for detecting *EML4-ALK* rearrangements (Kim et al., 2011). This diagnostic test offers clinicians a standardized, validated method to identify those patients more likely to benefit from therapy with crizotinib (Xalkori).

## Pharmacology and Pharmocokinetics

Crizotinib (PF-02341066) is an orally available multiple tyrosine kinase receptor inhibitor of ALK, hepatocyte growth factor receptor (HGFR, c-Met), and recepteur d’origine nantais (RON). In animal models, crizotinib exhibits concentration-dependent inhibition of ALK and c-Met phosphorylation. Following a single dose of crizotinib, the median time to peak concentration (C_max_) is reached within 5 to 6 hours and the mean bioavailability is 43%. With repeated doses of 250 mg twice daily, steady-state plasma concentration is reached within 15 days. Increasing pH decreases the solubility of crizotinib, and bioavailability may be reduced with coadministration of drugs that increase gastric pH. Although high-fat meals reduce the area under the curve and C_max_ by an estimated 14%, crizotinib may be given with or without food (Pfizer, 2011).

Crizotinib is primarily metabolized by CYP3A4 and CYP3A5 liver isoenzymes and is a time-dependent inhibitor of CYP3A4 and P-glycoprotein (P-gp). In vitro observation suggests that crizotinib is highly protein bound (91%). The metabolism of a single dose was affected by CYP3A4 inhibitors or inducers, but the effect on steady-state exposure remains to be evaluated. After consuming a single 250-mg dose of crizotinib, the mean terminal half-life was 42 hours. Clearances decrease at steady state due to the autoinhibition of CYP3A by crizotinib after multiple dosing (Pfizer, 2011).

The pharmacokinetic parameters of this chemotherapeutic agent do not appear to be affected by renal impairment, as steady-state concentrations in patients with mild and moderate renal insufficiency were similar to those in patients with normal renal function. For patients with hepatic impairment, plasma concentrations of crizotinib are likely to be increased, but data in patients with hepatic impairment are not available. Patients with total bilirubin > 1.5 × upper limit of normal (ULN) and alanine aminotransferase (ALT)/aspartate aminotransferase (AST) > 2.5 × ULN were excluded from clinical trials (Pfizer, 2011).

## Clinical Development

A phase I dose-escalation trial evaluating crizotinib as an oral single agent was initiated in May 2006. Thirty-seven patients with advanced cancer, including colorectal, pancreatic, sarcoma, anaplastic large cell lymphoma, and NSCLC, were enrolled in the study (Kwak et al., 2009). The maximum tolerated dose (MTD) was determined to be 250 mg twice daily. Observed dose-limiting toxicities were grade 3 increase in ALT and fatigue (Kwak et al., 2009). Responses for patients with NSCLC were most notable. Among the 10 NSCLC patients whose tumors were positive for the *EML4-ALK* gene rearrangements, 1 patient had a partial remission (PR), 2 patients achieved unconfirmed PR, and 4 patients had stable disease (Kwak et al., 2009).

The results of this phase I study led to subsequent clinical trials of crizotinib in the NSCLC patient population. There were two multicenter, single-arm studies (studies A and B) in which patients with ALK-positive NSCLC received crizotinib 250 mg twice daily (Camidge et al., 2011; Crino et al., 2011; Kwak et al., 2010). Patients included in both studies had received prior systemic therapy, except for 15 patients in study B who had no prior systemic treatment. In study A, 136 patients were identified as ALK-positive using the Vysis ALK Break Apart FISH Probe Kit. ALK-positive NSCLC was identified in the 119 patients in study B using various local clinical trial assays. The baseline patient characteristics were similar between the studies. The primary efficacy endpoint was objective response rate (ORR), defined as complete responses and partial responses, in both studies, using the Response Evaluation Criteria in Solid Tumors to assess disease response (Camidge et al., 2011; Crino et al., 2011; Kwak et al., 2010).

## Trial Results 

In study A, there were 67 partial responses and 1 complete response, with an ORR of 50% (95% confidence interval [CI]: 42%–59%). During the first 8 weeks of treatment, tumor response was achieved in 79% of the patients, and the median duration of response was 41.9 weeks (Crino et al., 2011). Among the 119 patients in study B, there were 69 partial responses and 2 complete responses, for an ORR of 61% (95% CI: 52%–70%). The estimated progression-free survival is reported at 10 months (95% CI: 8.2–14.7 mo). Tumor response was seen in 55% of the patients during the first 8 weeks of treatment, and the median duration of response was 48.1 weeks at the time of data cutoff (Camidge et al., 2011). The FDA’s approval of crizotinib was based on the efficacy data from these two studies.

## Indication

Crizotinib received accelerated FDA approval in August 2011. Crizotinib is indicated for the treatment of patients with locally advanced or metastatic NSCLC that is ALK-positive as detected by the FDA-approved FISH probe assay. This indication is based on response rate, as there are no data available showing improvements in patient-reported outcomes or survival (Pfizer, 2011).

## Dosing and Administration

The recommended dose of crizotinib is 250 mg taken orally twice daily with or without food. Therapy should be continued until clinical benefit is no longer seen. Dose reduction or interruption may be required based on patient tolerability. Crizotinib is available as 200-mg and 250-mg capsules (Pfizer, 2011). Patients must use specialty pharmacies to get crizotinib prescriptions filled.

Crizotinib has not been studied in patients with hepatic impairment, AST or ALT levels greater than 2.5 × ULN or greater than 5 × ULN if due to liver metastases, and/or total bilirubin greater than 1.5 × ULN. Since crizotinib is extensively metabolized in the liver, caution should be used in patients with hepatic impairment. With a lack of pharmacokinetic data, caution also needs to be used when prescribing crizotinib in patients with severe renal insufficiency or end-stage renal disease (Pfizer, 2011).

## Safety

Adverse reactions were reported from the two clinical studies discussed previously. In study A, dosing interruptions occurred in 36% of patients and lasted more than 2 weeks in 13% of the patients. In study B, dose interruptions occurred in 45% of the patients and lasted more than 2 weeks in 19% of the patients. Treatment-related adverse events leading to discontinuation of crizotinib therapy occurred in 6% of patients in study A and 3% of patients in study B. The most common adverse event was the occurrence of vision disorders, reported in 62% of patients in clinical trials; these disorders frequently occur within the first 2 weeks of treatment. They usually manifest as visual impairment, photopsia, blurred vision, vitreous floaters, photophobia, and diplopia. Patients experiencing visual symptoms should receive an ophthalmologic exam, especially because worsening symptoms can be indicative of more serious complications, including a retinal hole or retinal detachment (Pfizer, 2011).

Other common adverse reactions (> 25%) from both studies included vision disorder, nausea, diarrhea, vomiting, edema, and constipation (Table 1). Grades 3/4 adverse reactions, including increases in ALT and neutropenia, occurred in approximately 4% of patients in both studies (Camidge et al., 2011; Crino et al., 2011).

**Table 1:**
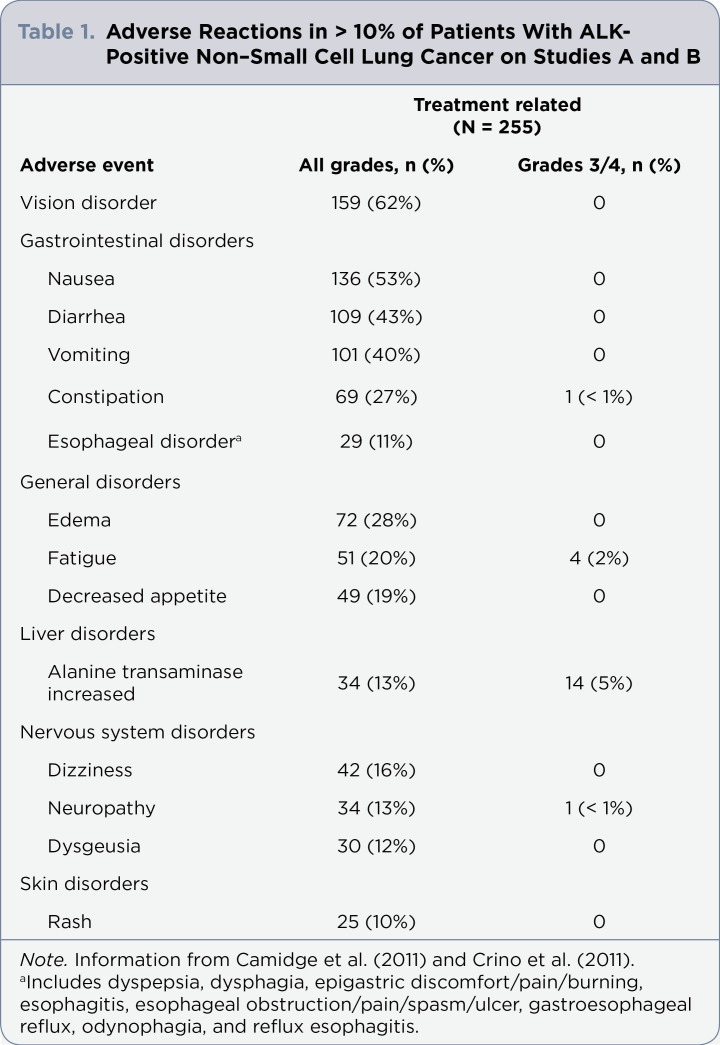
Table 1 Adverse Reactions in > 10% of Patients with ALK-Positive Non-Small Cell Lung Cancer on Studies A and B

Three adverse events listed by the manufacturer as “warnings and precautions” are pneumonitis, hepatic laboratory abnormalities, and QT interval prolongation (Pfizer, 2011). Potential life-threatening and treatment-related pneumonitis occurred in 4 of the 255 patients (1.6%) in clinical trials, all within 2 months of starting crizotinib therapy (Pfizer, 2011).

Grades 1/2 bradycardia was observed in 5% of patients. Patients with congestive heart failure, bradyarrhythmias, or electrolyte imbalances, or those who are taking medications known to prolong the QT interval, should be monitored for potential QTc prolongation. Grades 3/4 elevations in ALT were seen in 7% of patients in study A and 4% of patients in study B. These grades 3/4 elevations were reversible upon dosing interruption, and patients resuming treatment at a lower dose did not have recurrence. Neuropathy was also reported in 13% of patients receiving crizotinib, but most of these events were of grade 1 severity. Other serious adverse events reported in 2% of the patients were pneumonia, dyspnea, and pulmonary embolism (Pfizer, 2011).

## Drug Interactions

Crizotinib undergoes extensive metabolism in the liver by the CYP3A4 and CYP3A5 enzymes, making it susceptible to many drug interactions. Agents that are strong CYP3A inhibitors or inducers will affect plasma concentrations of crizotinib (Pfizer, 2011). Specific drugs that interact with crizotinib are included in Table 2.

**Table 2:**
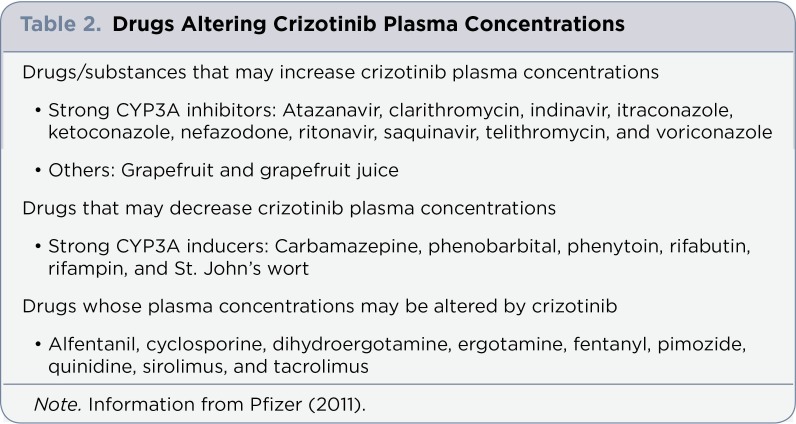
Table 2. Drugs Altering Crizotinib Plasma Concentrations

## Mechanism of Resistance

Initial response rates to crizotinib therapy were reported to be between 50% and 61% (Camidge et al., 2011; Crino et al., 2011). Although many patients experience substantial clinical benefit from crizotinib, the development of drug resistance has impacted its role in this disease. Similar to acquired resistance seen with other targeted therapies, including EGFR mutant lung cancers and v-raf murine sarcoma viral oncogene homolog B1 (BRAF) mutant melanomas, there are case reports demonstrating acquired resistance to crizotinib (Katayama et al., 2011).

Choi et al. (2010) reported two secondary point mutations within the kinase domain of the EML4-ALK in tumor cells isolated from a patient receiving treatment with an ALK inhibitor. These mutations, upon further examination, developed independently within subclones of the tumor. Researchers hypothesize that various amino acid substitutions at the gatekeeper position (T790M) of the ALK fusion protein could be a mechanism for resistance (Choi et al., 2010). In this patient who developed resistance to crizotinib after 5 months of treatment, molecular analyses showed the tumor had two acquired mutations within the kinase domain of EML4-ALK, C1156Y, and the gatekeeper mutation L1196M (Katayama et al., 2011). In vitro assays have also demonstrated L1196M gatekeeper mutation to be the major mechanism for crizotinib resistance (Ou, 2011). Another mutation, F1174L, has been identified in a patient with inflammatory myofibroblastic tumor and RANPB2-ALK translocation who relapsed on crizotinib therapy (Ou, 2011).

The exact structural basis for the crizotinib resistance is not entirely understood (Sasaki et al., 2010a). With the identification of the gatekeeper mutation, several second-generation ALK inhibitors are currently (or soon to be entered into) in clinical development to overcome resistance to crizotinib. One compound in clinical studies is AP26113, a selective small-molecule inhibitor of ALK. AP26113 has a fivefold greater potency than does crizotinib in inhibiting ALK as well as the L1196M gatekeeper mutation (Katayama et al., 2011). In a recent phase II study, another compound, Hsp90 inhibitor, showed activity against ALK-positive NSCLC patients. Hsp are chaperone proteins involved in stabilizing the EML4-ALK fusion protein in the cytoplasm (Ou, 2011). The link between Hsp90 and EML4-ALK can be interrupted by an Hsp90 inhibitor. When Hsp90 is inhibited, this results in degradation of EML4-ALK, leading to cell death in ALK-dependent cell lines (Ou, 2011). Early studies show that Hsp90 inhibitors lead to cell death in crizotinib-resistant tumors (Katayama, 2011).

## Implications for the Future

Although the subgroup of NSCLC patients with EML4-ALK translocation is small, the approval of crizotinib is a major breakthrough for individualizing anticancer therapy. Crizotinib received its accelerated approval based on early clinical trials demonstrating that patients with EML4-ALK–positive NSCLC have a high sensitivity to ALK kinase inhibition. Crizotinib was initially described in 2006, and by 2010 the first clinical trial results reported promising results in NSCLC patients carrying the ALK translocation (Kwak et al., 2010). The FDA granted approval in 2011 based on two clinical trials that included 255 patients with response rates of 50% and 61% (Camidge et al., 2011; Crino et al., 2011). Further trials will need to be conducted to assess crizotinib’s impact on overall survival in NSCLC.

Challenges still remain for the future, especially with using ALK inhibitors in combination with other agents. Currently there are two ongoing randomized phase III trials comparing crizotinib and chemotherapy. One trial, PROFILE 1014 (NCT01154140), is comparing crizotinib with platinum/pemetrexed (Alimta) combination chemotherapy in previously untreated patients with advanced ALK-positive NSCLC. Progression-free survival is being measured as the primary endpoint. PROFILE 1007 (NCT00932451) is comparing crizotinib with pemetrexed or docetaxel in the second-line setting in patients with ALK-positive NSCLC. The primary endpoint in this trial is progression-free survival as well.

For years, the treatment for advanced NSCLC included chemotherapy regimens that had limited effect. The approval of crizotinib offers a step forward in the treatment of patients with ALK-positive NSCLC. With continuous efforts to understand the molecular pathology behind NSCLC, many new molecularly targeted therapies are on the horizon.
